# Ureteroscopy and cystoscopy training: comparison between transparent and non-transparent simulators

**DOI:** 10.1186/s12909-015-0380-8

**Published:** 2015-06-02

**Authors:** Wen-Gang Hu, Jia-Yu Feng, Jin Wang, Ya-Jun Song, Xiao-Ting Xu, Hong Zhou, Chi-Bing Huang

**Affiliations:** Department of Urology, Second Affiliated Hospital, Third Military Medical University, XinQiao Street, ShaPingBa, Chongqing 400037 People’s Republic of China

**Keywords:** Simulation, Ureteroscopy, Cystoscopy, Urinary tract, Medical education, Training

## Abstract

**Background:**

Simulators have been widely used to train operational skills in urology, how to improve its effectiveness deserves further investigation. In this paper, we evaluated training using a novel transparent anatomic simulator, an opaque model or no simulator training, with regard to post-training ureteroscopy and cystoscopy proficiency.

**Methods:**

Anatomically correct transparent and non-transparent endourological simulators were fabricated. Ten experienced urologists provided a preliminary evaluation of the models as teaching tools. 36 first-year medical students underwent identical theoretical training and a 50-point examination of theoretical knowledge. The students were randomly assigned to receive training with the transparent simulator (Group 1), the non-transparent simulator (Group 2) or detailed verbal instruction only (Group 3). 12 days after the training session, the trainees’ skills at ureteral stent insertion and removal were evaluated using the Uro-Scopic Trainer and rated on an Objective Structured Assessment of Technical Skills (OSATS) scale.

**Results:**

The new simulators were successfully fabricated in accordance with the design parameters. Of the ten urologists invited to evaluate the devices, 100 % rated the devices as anatomically accurate, 90 % thought both models were easy to use and 80 % thought they were good ureteroscopy and cystoscopy training tools. The scores on the theoretical knowledge test were comparable among the training groups, and all students were able to perform ureteral stent insertion and removal. The mean OSATS scores of groups 1, 2 and 3 were21.83 ± 3.64, 18.50 ± 4.03 and 15.58 ± 2.23 points, respectively, (*p* = 0.001).

**Conclusions:**

Simulator training allowed students to achieve higher ureteroscopic and cystoscopic proficiency, and transparent simulators were more effective than non-transparent simulators.

## Background

The use of simulators in medical education began in the 1960s with devices for training resuscitation, anesthetic and clinical skills [1, 2]. Numerous studies have confirmed that medical students can improve their skills and achieve proficiency through simulation training [3–7].

Ureteroscopy and cystoscopy are essential for the diagnosis and therapy of urological diseases, and repeated hands-on training and standardized learning are necessary for mastering the skills required. However, due to ethical and fiscal concerns, traditional hands-on training of new practitioners with patients has been supplanted by methods that rely on endourological simulators [8, 9].

Both virtual and physical ureteroscopic and cystoscopic simulators have been created, and their value for training new students in the required surgical skills has been demonstrated [6, 10, 11]. Virtual models include the virtual-reality simulator for ureteroscopy [10] and the virtual reality endourological simulator [6]. However, the popularity of virtual models is limited by their high cost and the lack of haptic feedback for the trainee that is provided by physical simulators and clinical experience [3].

Current physical simulators are primarily bench models, such as the Uro-Scopic Trainer [11] and the adult ureteroscopy and renoscopy simulator [3]. Despite the relative fidelity of these simulators, novices often report that they cannot successfully relate the computer display to actual conditions encountered during surgery. The disparity between the simulated experience and reality encountered in clinical practice translates into greater risk of surgical error and a longer training period.

It seems logical that simulators fabricated from transparent material could help alleviate the shortcomings of the training methods described above. A transparent model could allow trainees to more readily observe and correct their errors during the course of training and self-evaluate their skills. To test this theory, we designed and fabricated both transparent and non-transparent ureteroscopy and cystoscopy simulators between October 1, 2013 and September 30, 2014. The present randomized, controlled trial study investigated the relative viability of these simulators as endourological training tools.

## Methods

### Physiological parameters

The physical dimensions of the simulator were chosen to conform to normal adult human anatomy (Table 1). Adult kidneys are paired organs that are typically 10–12 cm long, 5–7 cm wide and 3 cm in the anteroposterior dimension [12, 13]. The superior pole of the kidney is broader and thinner than the inferior pole [12] and the anterior surface is more convex than the posterior surface [14]. The thickness of the external renal cortex is 0.8 cm [15], and each kidney has 5–14 papillae, 8–18 minor calyces, 3 major calyces and 1 renal pelvis [16]. The ratio of papillae draining into minor calyces is 1:1–3:1 [15].

**Table 1:** Parameters of the endourological simulator

	Shape and physiological parameters
Kidneys	Bean-shaped, the superior pole is broader and thinner, the anterior surface is more convex; 12 cm in length, 7 cm in width, 3 cm in the anteroposterior dimension, 1 cm in thickness for renal parenchyma; 7 papillae, 7 minor calyces, 3 major calyces and 1 renal pelvis.
Ureters	27 cm (length), 7 mm (diameter), and 2.5 mm (thickness of the ureter wall) and 5 cm (distance of ureteral openings in bladder).
Bladder	Pyramid-like shape, the BWT (bladder wall thickness): 5 mm, the bladder volume: 300 mL, the intravesical height, depth and width: 5.5 cm, 10 cm and 9 cm, respectively.
Prostate	Conical in shape, wider at the top and tapering towards the base, 4 cm transversely at the base, 2 cm in its anteroposterior and 3 cm in its vertical diameter.
Male urethra	A double curve was set, the posterior urethra: 5 cm in length, the anterior urethra: 15 cm in length, the mean diameter: 9 mm, the thickness of its wall: 5 mm, the distance between the penis neck and the external urethral orifice: 1.5 cm, the thickness of glans wall: 7 mm.
Female urethra	4 cm in length and 9 mm in diameter.

Humans have two ureters, thick-walled muscular tubes that each measure 25–30 cm in length [16] and 1–10 mm in diameter [12]. The ureteric openings are about 5 cm apart in the distended bladder of both genders [13].

The bladder is somewhat pyramidal in shape when empty [14]. The average thickness of the bladder wall is about 3.0 ± 1.1 mm in the adult male and 3.0 ± 1 mm in the adult female [17, 16]. The mean capacity of the adult male bladder is slightly more than that of the female bladder at 220 mL, and varies from 120 to 320 mL. Bladder volume is calculated as: bladder volume = bladder height × bladder depth × bladder width × 0.6 [18].

The prostate is somewhat conical in shape, wider at the top and tapering towards the base with the urethra passing through the center, and measures approximately 4 cm transversely at the base, 2 cm at the anteroposterior end and 3 cm in vertical diameter [13].

The male urethra extends from the internal orifice of the urinary bladder to the external opening. It can be subdivided into the posterior and anterior urethra. The posterior urethra is 3–5 cm long, while the anterior urethra is 15–20 cm long [12, 19]. The urethra has a double physiological curve. The first curve (infrapubic) is constant unless exposed to a strong force, and the second curve (praepubic) can be eliminated by a tiny force and vanishes naturally during an erection [12, 16]. Ouattara [20] reported that the mean urethral diameter can reach 11 to 15 mm and that the average thickness of the periurethral tissue is 9 mm. Additionally, there is a narrowing of the urethra (the penis neck) at the base of the glans. The female urethra is straight, about 4 cm long and 6 mm in diameter [16].

### Design, fabrication and assembly of the transparent simulator

The simulator was designed in accordance with three criteria: its dimensions should conform to human anatomy; it should be composed of transparent materials; and it should be able to satisfy the requirements of typical ureteroscopy and cystoscopy training. Training procedures included guidewire insertion, ureteral stent insertion/removal and stone extraction.

**Fig. 1:**
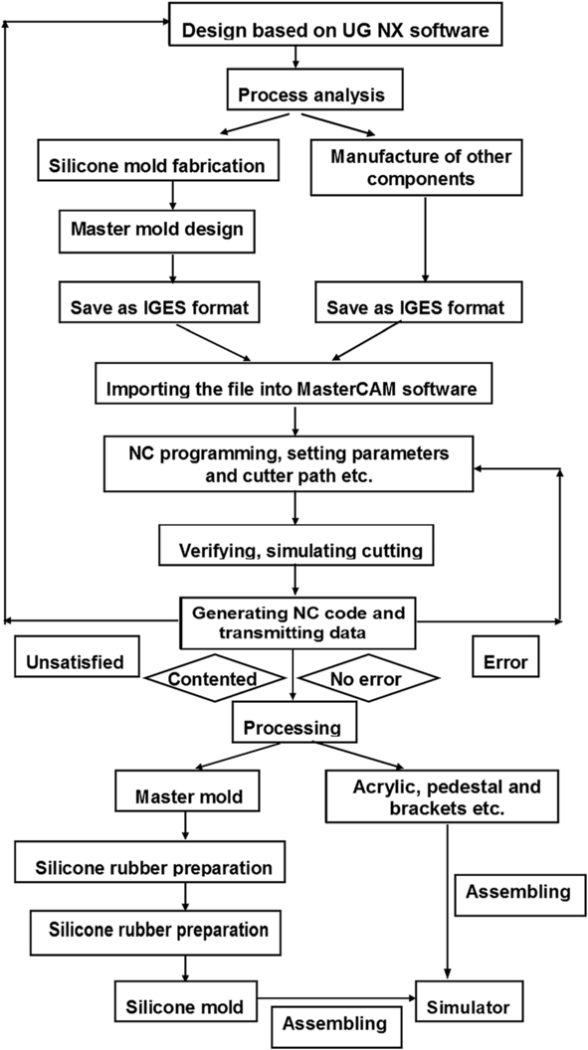
Flow diagram of the general approach for creating the transparent simulator

**Fig. 2:**
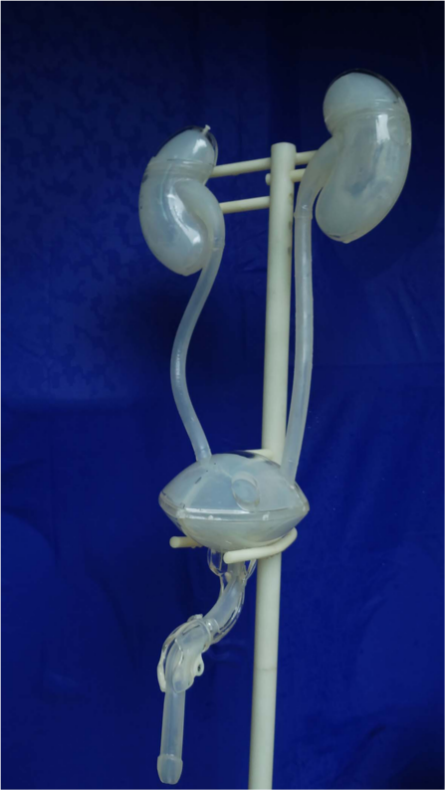
The assembled transparent simulator, configured to simulate male urinary anatomy

**Fig. 3:**
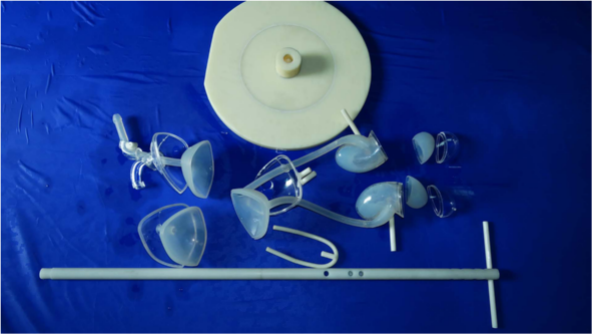
All components of the transparent simulator: kidneys, ureters, bladder, urinary tracts, brackets and base

The equipment used in the design and fabrication of the transparent simulator consisted of UG NX software (Version 7.5, Siemens, Plano, TX, USA), MasterCAM software (Version 9.1, CNC Software, Tolland, CT, USA) and a computer numerical control machine (JingYi, China), The model was fabricated (Fig. 1) from the following materials: silicone rubber, transparent poly-methyl-methacrylate acrylic (P-M-MA) and acrylonitrile butadiene styrene resin (ABS; all from Fushengyuan, Dongguan, China). The complete, assembled transparent simulator and the unassembled components of the simulator are pictured in Figs. 2 and 3, respectively.

### Fabrication of the non-transparent simulator

To obtain a non-transparent simulator, a transparent simulator was spray-coated with a layer of black paint on its exterior surface. Otherwise, the transparent and non-transparent simulators were identical in terms of structure, function and operational approaches.

### Preliminary evaluation

The Ethics Committee of Second Affiliated Hospital of Third Military Medical University of China approved all stages of the simulator evaluation plan. Ten urologists (seven men and three women) were selected from 100 urologists affiliated with the Third Military Medical University through stratified sampling and were invited to evaluate the simulators.

The urologists were given an introductory presentation of the simulator, including an explanation of its structure and operation. They were then asked to use both the transparent and non-transparent simulators for about 30 min, to perform separate simulated cystoscopy, ureteral stent insertion and removal and removal of upper urinary tract stones. After this, each urologist was asked to complete a standard anonymous questionnaire assessing both simulators. The questionnaire had three questions: “Do you think the simulator is anatomically accurate?”; “Do you think the simulator is easy to use?” and “Do you think the simulator is a good practice format?”. The participants were asked to answer “yes” or “no” to each question.

### Validity assessment design

Thirty-six first-year medical students were recruited from Third Military Medical University through a recruitment conference to assess the viability of the simulators for teaching endoscopic skills. Each recruited student verbally consented to participate in the study and signed a consent form. All students attended the same 1-day didactic lecture given by an endourologist, and two six-hour days of video instruction encompassing genitourinary anatomy, cystoscopy, ureteroscopy and ureteroscopic procedures, ureteral stent insertion and removal by transurethral cystoscopy and stone extraction by transurethral cystoscopy or ureteroscopy. A 50-point examination paper designed by the endourologist lecturer and based on the course content was administered to test the students’ theoretical knowledge the next day. The examination paper consisted of 50 multiple-choice questions, and each question on the exam had just one right answer.

**Fig. 4:**
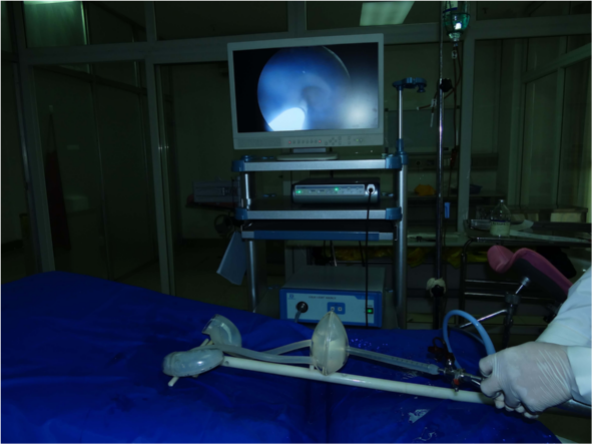
Ureteral stent insertion in the transparent simulator

**Fig. 5:**
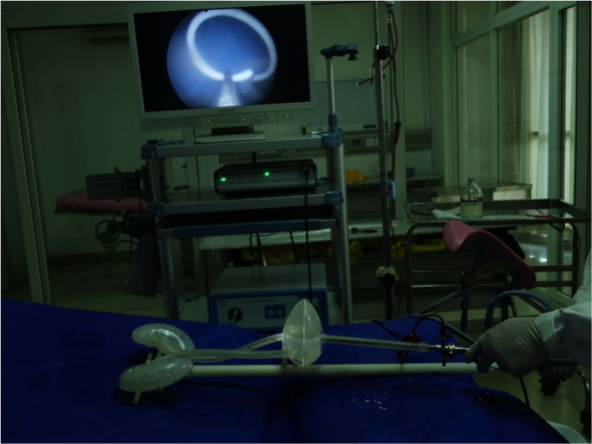
Ureteral stent removal in the transparent simulator

The day after the exam, students were randomly assigned to three groups. Random assignment was conducted using a random number table, and each group had 12 participants. Students used the transparent simulator in the experimental group (Group 1) and the non-transparent simulator in the experimental control group (Group 2). Within each group, students were further separated into working pairs, with one student operating and the other assisting. Under the supervision of an experienced instructor, students repeatedly practiced cystoscopy, guidewire insertion, ureteral stent insertion and removal, ureteroscopy and stone extraction using the simulator. Each 2-person team spent approximately one hour per day training on the simulator. In both groups, each student participated in 12 one-hour training sessions. Ureteral stent insertion training and ureteral stent removal training are pictured in Figs. 4 and 5, respectively.

Students were also grouped into pairs in a control group (Group 3) that received no simulator training. Instead of simulator training, students were given verbal instruction concerning the proper performance of cystoscopy, ureteroscopy, guidewire insertion, stone extraction and ureteral stent insertion and removal. The control group was taught by an experienced instructor in the field of cystoscopy and ureteroscopy. The total instruction time for Group 3 was equal to the hands-on training time of the experimental groups, but no hands-on training was conducted.

During the training period, a student-centered educational approach was emphasized. The students themselves planned and managed their own learning. The instructors were responsible for finding and correcting the students’ errors, as well as answering the trainees’ questions. Additionally, the instructor gave a daily demonstration of the ureteroscopy and cystoscopy operation for all participants of each group prior to the start of training or verbal instruction.

### Qualitative assessment

The day after the conclusion of the 12-day training period, the proficiency of all trainees at ureteral stent insertion and removal was evaluated using an Uro-Scopic Trainer (Limbs & Things, Bristol, UK). The commercial simulator consists of a model of the male genitourinary tract that can allow the standard instruments to pass. An Objective Structured Assessment of Technical Skills (OSATS) global rating scale was used to assess their ability to perform endoscopy. The first step of the student evaluation was a surgery-specific checklist that included the following five steps (Table 2): assembly of the cystoscope, correct positioning of the cystoscope at the ureteral opening in the bladder, accessing the ureter with a guidewire and ureteral stent insertion and removal. One point was awarded for each item that was completed.

**Table 2:** Operation-specific checklist

	Not done or incorrect	Done correctly
Cystoscope assembling	0	1
Positioning ureteral opening in the bladder	0	1
Accessing the ureter with a guidewire	0	1
Ureteral stent insertion	0	1
Ureteral stent removal	0	1
Total		

The second step of the student evaluation used the OSATS (Table 3) to assess the quality of the trainee’s performance. OSATS is a reliable and valid tool for the assessment of technical skill, and can be used to measure clinical competence [21, 22]. During this period, student performance was independently evaluated by two experienced urologists and given a score of 1 to 5. This assessment involved seven criteria: tissue handling, time and motion, instrument handling, knowledge of the instruments, flow of surgery, use of assistants and knowledge of the procedure. The maximum score a trainee could achieve was 35 points. The recorded score of every participant’s performance was the mean of the scores given by the two evaluating urologists.

**Table 3:** The global rating scale used to assess the quality of the students’ performance

Global rating scale of operative performance					
Please circle the number corresponding to the candidate’s performance in each category, irrespective of training level.					
	1	2	3	4	5
Respect for tissue	Scope frequently pushed into urothelial wall. Used unnecessary force with guidewire.	Scope occasionally pushed into urothelial wall. Used unnecessary force with guidewire.	Careful handling of tissues, but occasionally caused inadvertent tissue damage.	Careful handling of tissues, but on one occasion caused inadvertent tissue damage.	No trauma to urothelial wall with scope. Consistently handled tissues appropriately.
Time and motion	Many unnecessary moves.	Occasional unnecessary moves.	Some unnecessary moves, but time more efficien.t	Efficient time/motion but one unnecessary move.	Clear economy of movement and time is maximized.
Instrument handling	Repeatedly makes tentative or awkward moves with instruments by inappropriate use of instruments.	Occasionally makes tentative or awkward moves with instruments by inappropriate use of instruments.	Competent use of instruments, but occasional stiff or awkward movements.	Used appropriate instruments, but made one awkward movement.	Fluid movements with instruments and no awkwardness.
Knowledge of instruments	Frequently asked for wrong instruments or used inappropriate instruments.	Occasionally asked for wrong instrument or used inappropriate instruments.	Knew the names of most instruments and used instruments appropriately.	Knew the names of the instruments, but used one inappropriately.	Obviously familiar with instruments and their names.
Flow of operation	Frequently stopped and seemed unsure of next move.	Occasionally stopped and seemed unsure of next move.	Demonstrated some forward planning with reasonable progression.	Demonstrated forward planning with only one unsure episode.	Well planned operation with effortless flow of movements.
Use of assistants	Frequently poorly placed or failed to use assistants.	Occasionally poorly placed or failed to use assistants.	Used assistants well most of the time.	Only once failed to use assistants.	Strategically used assistants to the best advantage at all times.
Knowledge of procedure	Needed specific instruction at all steps.	Needed specific instruction at most steps.	Knew all important steps, but needed one instruction.	Knew all important steps of operation.	Familiar with all aspects of operation.

Overall of circled numbers for all rows

### Statistical analysis

Two researchers independently conducted the statistical analyses using SPSS software, version 17.0 (SPSS, Chicago, IL, USA). The differences between the groups’ scores on the theoretical test and the qualitative assessment of performance were compared using one-way analysis of variance and the least significant difference test. Statistical significance was accepted at *p* < 0.05.

## Results

The simulators were successfully developed and fabricated. Ten urologists were recruited and voluntarily participated in the simulator evaluation. Two of the ten participants (both men) were chief physicians, two (both men) were associate chief physicians, three (two men and one woman) were attending physicians and three were residents (one man and two women). The average age of the evaluating urologists was 38.55 years (SD 8.78; range 27 to 55) and the average working age was 13.80 years (SD 8.18; range 3 to 32). All of the volunteers agreed to have their data included in this analysis. Of the 10 participating urologists, 100 % rated the simulators as anatomically accurate, 90 % thought they were easy to use and 80 % thought they could serve as ideal training devices.

Thirty-six eligible first-year medical students were recruited to assess the viability of the simulators for teaching endoscopic skills. None of the students elected to have their data withheld from the study. There were no significant differences in the 50-point theoretical knowledge test scores of Group 1, Group 2 and Group 3 (41.92 ± 2.84, 40.67 ± 3.08, 39.92 ± 3.60, respectively, *p* = 0.312 > 0.05; Table 4). All the students performed the five steps of ureteral stent insertion and removal correctly, and each group received the full five points for this stage of the evaluation.

**Table 4:** Theoretical knowledge test and performance assessment scores^a^

		Theoretical knowledge test	Assessment of performance
Group 1	Transparent model-based training	41.92 ± 2.84 (38–47)	21.83 ± 3.64 (15–27)
Group 2	Non-transparent model-based training	40.67 ± 3.08 (36–45)	18.50 ± 4.03 (13–26)
Group 3	No training	39.92 ± 3.60 (35–46)	15.58 ± 2.23 (12–20)
P-values	Overall	0.312	0.001
	Group 1 cf. Group 2	0.343	0.022
	Group 1 cf. Group 3	0.133	0.001
	Group 2 cf. Group 3	0.568	0.043

^a^The five steps of the procedure were completed by each group

In the performance quality assessment, Groups 1, 2 and 3 achieved mean scores of 21.83 ± 3.64, 18.50 ± 4.03 and 15.58 ± 2.23 points, respectively (*p* = 0.001). Multiple comparison analysis showed that the mean score of Group 1 was significantly higher than that of Group 2 (*p* = 0.022), and that the mean score of Group 2 was significantly higher than that of Group 3 (*p* = 0.043). The interrater reliability of the OSATS scores between the two raters was high. The value of Spearman’s rho was 0.823.

## Discussion

A training deficit exists in contemporary medicine that can have severe and even deadly consequences. The reasons for this deficit include fiscal constraints [23], lack of clinical training positions, a rapidly increasing number of medical students and intense doctor-patient conflicts [24, 25]. Consequently, the opportunities for undergraduates or postgraduates to participate in programs for the training of clinical skills are severely limited, and junior doctors complete their training without becoming sufficiently acquainted with surgical procedures.

Much research has been conducted seeking effective methods of improving operational skill training [26, 27, 3, 7]. Many of these studies have concluded that the development of improved training simulators and simulation training programs are promising methods for improving access to hands-on clinical skills training. However, Matsumoto *et al.* [28] conducted a study comparing a low fidelity endourological bench simulator with a high fidelity model, with regard to the endourological proficiency of trainees. The low fidelity simulator was assembled from a Penrose drain (urethra), an inverted Styrofoam cup (bladder), molded latex and two embedded drinking straws (ureters). The investigators concluded that novices trained on the two simulators achieved a similar level of skill improvement, and that the key to the design of bench models was the identification of essential constructs rather than the accurate representation of anatomy.

Furthermore, Chou *et al.* [10] concluded that a virtual reality simulator was as effective as a bench simulator for teaching basic ureteroscopy skills to inexperienced trainees. The virtual reality simulator would also eliminate recurring costs, require minimal instructor input and could drastically reduce training costs once the initial training protocol was established. Shashikant *et al.* [11] compared the use of a live porcine model with a virtual reality simulator and determined that the overall usefulness of the models were similar. Endourological training using a live porcine model was found to be more realistic, but the virtual model was more feasible for repetitive tasking [11]. Despite the advantages of virtual simulators, they lack haptic feedback and trainees report that they do not always effectively simulate the actual conditions encountered during surgery.

To our knowledge, no studies have been conducted that investigate the effectiveness of transparent simulators for training endourological skills. This study reports the relative effectiveness of using transparent anatomic models compared with non-transparent models for teaching proper ureteroscopic and cystoscopic techniques.

In the preliminary evaluation, we recruited 10 urologists to evaluate the simulators. The urologists determined that the simulators were anatomically accurate, easy to use and could potentially serve as ideal training devices.

We then recruited 36 first-year medical students who were given training with the transparent simulator (Group 1), the opaque simulator (Group 2) or no practical training (Group 3). The groups received identical initial instruction by an expert lecturer and subject matter videos. There were no significant differences among the three groups in their theoretical knowledge, according to the results of a 50-point exam given at the conclusion of the instruction. Consequently, the significant differences in performance scores of the three groups at the conclusion of simulator training indicated that, compared with verbal instruction alone, both transparent and non-transparent simulator training resulted in higher cystoscopy and ureteroscopy proficiency. Furthermore, the transparent simulator was more effective in training endoscopy techniques than the opaque simulator. Our results are consistent with those reported by other researchers [3–7].

The main factor contributing to the significant difference in procedural skills between students trained with the transparent and opaque models was likely the ability of the former to observe internal anatomical structure and the various procedural stages while training. The use of optically transparent materials was key to the success of the simulator, because it provides immediate visual feedback for the trainee during the procedure. The transparent model enhances and reinforces the trainee’s recall of the surgical approach, and errors are immediately apparent that would be concealed by an opaque device. Additionally, the use of transparent materials permits the tutor to better observe the operation and provide students with immediate and relevant feedback.

A potential concern regarding the transparent simulator may be that transparent materials are prohibitively more costly. However, this is not the case. Optically transparent materials are widely used industrially and in daily life, and the cost of these materials is no more expensive than that of any common plastic. The acrylic used to manufacture our device cost ~ $60 per kilogram, whereas the common opaque plastic ABS resin that might have been used to manufacture an equivalent opaque device costs ~ $50 per kilogram. The discrepancy between the prices of the transparent and the non-transparent materials in the manufacture of each simulator is almost negligible.

A number of limitations of the present study should be considered. The differences between the no training and model-based training groups were relatively small, and the scores of the intervention groups were not high. The relatively low scores may have been due to insufficient training time and trainee unfamiliarity with the examination simulator; therefore, a longer training period and increased trainee familiarity with the commercial simulator might have improved scores. However, whether increased training time and trainee familiarity with the simulator would have increased or decreased the differences between the different trainee groups requires further investigation. Additionally, the silicone rubber used to fabricate the model was not perfectly transparent. It is possible that improved transparency of the training simulator could further improve the final assessment of procedural skills. Therefore, future devices may make use of other materials, such as hydrogels, that have better transparency. Finally, our study does not address whether the tutors were satisfied with the transparent simulator as a teaching tool. Yet, there is no denying that the instrument was helpful for the trainees. In view of these limitations, additional studies should be conducted, including studies that extend training time, explore the use of different transparent materials and evaluate the efficacy of the simulator from a teaching perspective.

## Conclusions

The use of a model simulator allowed trainees to achieve higher ureteroscopy and cystoscopy proficiency compared with verbal instruction alone, and the transparent simulator was more effective than the non-transparent device. Transparent simulators are potentially powerful training tools that may also be useful in relevant research.

## Abbreviations

OSATS: Objective structured assessment of technical skills
P-M-MA: Poly-methyl-methacrylate acrylic
ABS: Acrylonitrile butadiene styrenep
SD: Standard deviation
